# Dimensions of Identity and Subjective Quality of Life in Adolescents

**DOI:** 10.1007/s11205-015-0942-5

**Published:** 2015-03-20

**Authors:** Maria Oleś

**Affiliations:** Institute of Psychology, John Paul II Catholic University of Lublin, Al. Racławickie 14, 20-950 Lublin, Poland

**Keywords:** Adolescence, Commitment, Exploration, Identity status, Quality of life

## Abstract

The aim of this study is to investigate the relations between identity statuses and the perceived quality of life in adolescents aged 16–19. The research methods include the Ego Identity Process Questionnaire to assess identity status of a sample covering 233 participants (148 girls, 85 boys), and the Youth Quality of Life Instrument to assess their subjective quality of life. Diffused identity is linked to the lowest level of subjective quality of life, whereas foreclosed identity to the highest. Five patterns of the connection between identity dimensions and perceived quality of life have been distinguished through cluster analysis. The results indicate that different patterns of identity processes in adolescents coexist with different levels of quality of life.

## Introduction

Adolescence is usually considered as a transitional period between childhood and adulthood. This period of life involves searching for and forming one’s own identity. Identity formation is a dynamic, long-lasting process when the young person tries to find answers to such essential questions as “Who am I?”, “What do I want to achieve?”, “Which values are important to me?”, “Who do I want to be in the future?”, “What is my position in the community?”, “How to build relationships with others?”, and “What choices to make?” (Erikson 1963; see also Arnett 2002; Beyers and Çok 2008; Steinberg 2008). According to Erikson (1968), identity provides frames of reference for interpreting individual experiences and allows one to define one’s own aims and directions in life. Resolving this identity crisis is influenced by social surroundings and individual development including the way of solving previous developmental challenges.

## Identity Constructs and Quality of Life Issues

### Identity: Current Status of Research

Identity crisis can be analyzed in two dimensions: Exploration and Commitment, distinguished by Marcia (1966). According to him, Exploration is a process leading to the choice of aims, values, roles and beliefs while Commitment is an engagement in a particular type of professional, interpersonal, religious and social activity resulting from solving identity issues (Marcia 1966; see also Berzonsky 2003). Both processes contribute to the differences in experiencing an identity crisis. Due to these two processes it is possible to distinguish four identity statuses: *Diffused*, *Foreclosed*, *Moratorium* and *Achieved*. To begin with, identity is diffused when the individual neither explores nor is engaged in goal/value directed activities in their life. Identity foreclosure, in turn, is a status of identity characterized by commitment without exploration of alternatives in contrast to moratorium which involves exploration without commitment. Finally, achieved identity is a status of identity typical of the individual who has experienced a crisis, undergone identity exploration and decided on their commitment. According to Marcia (1967), individuals with achieved identity are more mature, with an internal locus of self-definition. In their later works, Luyckx et al. (2005) empirically derive identity statuses similar to those described by Marcia (1966). They unpack both Commitment and Exploration, each into two forms, and they distinguish the following four identity dimensions: *Commitment Making*, *Identification with Commitment, Exploration in Breadth,* and *Exploration in Depth.* Commitment making applies to choice-making. Identification with commitment refers to the degree of identification with the choices. Exploration in breadth concerns collecting information on different possibilities to guide the choices one makes. Exploration in depth refers to gathering information on current choices to guide the maintenance and evaluation of these choices. These statuses, or types, are associated with adjustment, well-being, and distress (Luyckx et al. 2006b). In the literature of the subject, there appear some complementary approaches to understanding identity development (Archer and Waterman 1990; Berman et al. 2000; Berzonsky and Adams 1999; Kroger and Marcia 2011; Schwartz 2001; Schwartz et al. 2000; Waterman 1999). Crocetti et al. (2008) introduced three factor model of identity: commitment, in-depth exploration, and reconsideration of commitment as basic identity processes. Within the framework of constructivism one may refer to the identity styles of Berzonsky (1990) who proposes a process-oriented paradigm of identity formation from a constructivist theoretical perspective. “Individuals construct both a sense of who they think they are and the ‘reality’ within which they act” (Berzonsky 1990, p. 156). He distinguishes three different cognitive orientations named identity styles by which the individual can evaluate, structuralize, revise and utilize self-relevant information. They include *Informational*, *Normative* and *Diffuse/Avoidant* style (Berzonsky 1993; see also Schwartz et al. 2000). An informational style represents a willingness to examine different solutions to a problem and to test diverse options before committing oneself to any of them. A normative style refers to growth by conforming to the expectations of social environment and a high degree of commitment to authority. A diffuse/avoidant style is characterized by the tendency to delay and to make decisions depending on situation (Berzonsky 1993). In the constructivist approach, the informational style may be seen as the most preferable way of identity formation. It probably corresponds to a greater openness to experience, to activity based on seeking information, consideration of different options, and the manifestation of great ego control (Schwartz et al. 2000). The next approach to identity formation, developed by Waterman (1990, 1992, 1993, 1999), aims at including identity statuses in the area of self-realization theory, e.g. humanistic personality theories and Aristotelian Eudaimonism. In the process of identity formation “a person’s sense of identity is an approximation of the daimon, a set of goals, values, and beliefs that, to a greater or lesser extent, correspond to the actual potentials of the individual” (Waterman 1992, p. 58). He distinguishes *Personal Expressiveness* versus *Instrumentality* as the third defining dimension of identity. This differentiation complements the dimensions of exploration and commitment developed by Marcia (1966; see also Schwartz et al. 2000). Although the construct of Waterman’s personal expressiveness and the paradigm of identity styles of Berzonsky are based on contrasting approaches, there is a connection between personal expressiveness and different identity styles (Berzonsky 1989). There are theoretical and empirical associations between the three conceptualizations of identity formation (Schwartz et al. 2000).

From an existentialist perspective, the co-constructivist approach of Kurtines (1999) conceptualizes identity emphasizing choice, moral-development, self-control and responsibility for promoting growth of society. Identity statuses are also described from the point of view of existentialist philosophy, while identity formation is further discussed from the feminist perspective (Bilsker 1992; Archer 1992).

### Subjective Quality of Life

Over the last two decades, one can observe an intensive progress of research on quality of life not only in the field of health care and social services but also in the relation to more specific variables such as personality traits, psychosocial adaptation, coping strategies, etc. Therefore, the construct of quality of life has increasingly become a focus for psychological research. According to WHOQOL approach, quality of life is “the individual’s perception of their position in life in the context of the culture and value systems in which they live and in relation to their goals, expectations, standards, and concerns. It is a broad-ranging concept affected in a complex way by the person’s physical health, psychological state, level of independence, social relationships, and their relationship to salient features in their environment” (The WHOQOL Group 1995, p. 1405). This definition emphasizes not only the subjective nature of quality of life, but also its cultural and environmental context as well as personal goals and values. At the same time, personal identity formation implies identification with specific values and choice of life goals and, moreover, this process is deeply rooted in socio-cultural context. Thus, searching for the relationship between identity status and quality of life is well-justified and particularly required as far as adolescents are concerned.

So far, the research on quality of life in adolescents has dealt with the issue of identity crisis—so crucial a developmental phenomenon—in a limited way only. Since a person’s way of functioning is different at every stage of identity formation, the following question arises: In what way is the subjective quality of life related to a particular level of identity development? In other words, what is the relationship between identity development and perceived quality of life?^1^

## Identity Issues and Quality of Life in Psychological Investigations

With specific reference to identity statuses distinguished by Marcia (1966), empirical research concerns the relation between them and psychosocial attitudes, personality traits, and behavioral variables. According to the results, identity diffusion is associated with emotion-focused coping, neuroticism, depression, poor adjustment, and, in a negative way, with self-awareness, cognitive persistence, conscientiousness, and well-being (Berzonsky and Ferrari 1996; Berzonsky 2003). Foreclosed identity is related to conscientiousness, agreeableness, well-being, and also to a limited tolerance for information threatening, personal beliefs or values. Achieved identity and moratorium are connected with the cognitive needs, self-reflection, problem-focused coping, openness, agreeableness, and conscientiousness (Nurmi et al. 1997; Berzonsky 2003). Moreover, achieved identity implies high self-esteem, autonomy, internal locus of control, well-being, a more mature ego and relatively fewer disorders (Cramer 1997). Persons in moratorium are characterized by a higher level of anxiety, conflicts with parents and a lower level of well-being. They tend to be depressed and anxious (Berman et al. 2006b; Kidwell et al. 1995), but also more creative in thinking (Berman et al. 2000). On the other hand, individuals in identity foreclosure have a higher level of authoritarianism (Marcia 1967), conventional moral thinking and conformism as well as high self-esteem and self-satisfaction (Marcia 1980), low autonomy and external locus of control. For diffused individuals, lower level of moral thinking and ego development, low level of self-esteem and autonomy, external locus of control, high anxiety, neuroticism, and low conscientiousness are characteristic (Waterman 1999; Cramer 1997). What is more, diffused persons are often apathetic (Marcia 1980) and have academic difficulties (Berzonsky 1985). While achieved adolescents have the highest level of adjustment and diffused subjects have the most negative profile of adjustment, moratorium and foreclosure adolescents score in between (Côté and Schwartz 2002).

The research conducted so far has examined the level of maturity among people representing particular identity statuses and the connection between adjustment and identity style (Berzonsky 1990). Identity styles result from socio-cognitive strategies used in coping with identity issues. People with moratorium or achieved identity usually use information orientation, whereas those with foreclosed identity—normative orientation, while those with diffused identity—diffuse/avoidant orientation. The orientation of the first type is considered as the most mature, the last one is the least mature, whereas normative orientation is located in the middle. According to Erikson (1968), formed identity is an expression of a healthy personality and is directly connected with well-being. This idea, taken up by Vleioras and Bosma (2005) who examine the relationship between identity styles and well-being, following Ryff’s model (1989), consists of self-acceptance, environmental mastery, positive relationships with others, purpose in life, personal growth and autonomy. Information orientation correlates positively with problem-focused and effective coping, and openness to experience. Diffuse/avoidant orientation corresponds negatively to the quality of relationships with peers, achievements at school and self-esteem, and in a positive way with non-adaptive decision-making strategies, drug and alcohol addictions, depressive reactions, neuroticism and behavioural and/or eating disorders (Berzonsky 1990, 1992, 2003; see also Vleioras and Bosma 2005). Well-being correlates positively with information orientation but negatively with diffuse/avoidant orientation. However, the correlation between normative orientation and well-being turns out to be unclear. The research results show negative connection between well-being and avoiding identity issues, yet when these issues are taken up, the way in which an individual approaches them is not important (Vleioras and Bosma 2005).

Some other correlates of identity have also been investigated, for instance, adjustment and ego development (Dunkel and Papini 2005; Erlanger 1998), attachment style (Berman et al. 2006a; Kennedy 1999), or defense mechanisms (Cramer 1995).

Phillips and Pittman (2007) examine the relationships between identity styles and well-being indicators as well as the attitudes toward the future in adolescents aged 11–20. The following indicators of well-being are taken into consideration: self-esteem, pessimism, approval of criminal activity, expectations concerning future education, and optimism/efficacy. As anticipated, people with a diffuse/avoidant orientation are characterized by a lower level of well-being, hope and self-esteem, and higher level of hopelessness with a greater approval of criminal activities than participants with an informational or normative orientation.

In the research conducted by Schwartz (2004), agentic personality scales including self-esteem, purpose in life, ego strength, internal locus of control differentiate significantly between identity achievement and foreclosure statuses on the one hand, and moratorium and diffused identity status on the other, in favour of those with achieved and foreclosed identity. Self-actualization is a variable differentiating between moratorium and achievement statuses on the one hand, and diffusion and foreclosure on the other, in favour of achieved identity and moratorium.

Crocetti et al. (2012) investigated links between identity configurations and internalizing problem behaviours in adolescents, relationships between identity configurations and identity functions in late adolescents and emerging adults, as well as associations between sense of coherence, and basic psychological need satisfaction in emerging adults. Chen and Yao (2010) investigated the relation between self-identity and health-related quality of life (HRQOL) in adolescence. The results demonstrated that the concept of identity firmness predicted adolescent’s HRQOL more than the concept of identity importance.

Summing up, the difficulties in identity formation in adolescents are related to the problems connected with psychosocial functioning which implies lowered quality of life, whilst identity achievement is connected with well-being.

## Purpose of the Study

The research problem of the present study addresses the following question: In what way does the level of subjective quality of life in adolescents depend on the stage of identity development? In other words, how do adolescents perceive their quality of life at different identity statuses as conceptualized by Marcia (1966): achievement, moratorium, foreclosure and diffusion and/or representing different degrees of exploration and commitment? For this purpose, three hypotheses have been formulated:

### **H1**

There are relationships between identity dimensions and subjective quality of life in adolescents: quality of life positively relates to commitment, and negatively to exploration.

### **H2**

The highest level of perceived quality of life reveals the achieved adolescents, and the lowest level of quality of life is the feature of the diffused ones.

### **H3**

There are differences in the level of subjective quality of life between groups of participants characterized by a different pattern of identity processes in reference to quality of life—both in the total score and in particular dimensions of quality of life. The expected pattern is: adolescents in the achieved status would be highest in quality of life.

## Methods

### Measures

#### Ego Identity

To assess identity status according to Marcia’s approach, the *Ego Identity Process Questionnaire* (EIPQ; Balistreri et al. 1995) is used. The EIPQ consists of 32 statements (12 with a reverse key) with a 6-point answer scale (from 1—“strongly disagree”, to 6—“strongly agree”). The questionnaire provides scores for exploration (E) and commitment (C). Identity status is determined on the basis of presence or absence of crisis and commitment in four ideological domains (politics, occupation, religion and values) and in four interpersonal domains (family, friendship, dating and gender roles). The participants obtain total scores for exploration and commitment separately, each of which can range between 6 and 96. To determine the identity statuses for each person, median scores are used. Participants above the median in both dimensions are classified as identity achieved, respondents below the median are classified as diffused. Those above the median in commitment but below in exploration are classified as foreclosed, however, respondents with reverse pattern are classified as moratorium. In the study of Balistreri et al. (1995) median scores of 66.5 for exploration and 62 for commitment are used. Psychometric properties of the EIPQ are satisfying; Cronbach’s alpha for scale E is, *α* = 0.86, for scale C, *α* = 0.80. Stability for scale E, equals *r*_*tt*_ = 0.76, and for scale C, *r*_*tt*_ = 0.90. The correlation between dimensions C and E is negative (*r* = −0.35, *p* < 0.05) (see also Luyckx et al. 2006a). Confirmation factor analysis confirms the two-factor structure of the questionnaire.

#### Quality of Life

The Polish version of the *Youth Quality of Life Instrument*—*Research Version* (YQOL-R; Patrick et al. 2002) is used to assess quality of life. The YQOL-R is a self-assessment method used for measuring perceived quality of life in youths aged 11–18. The scale consists of 41 statements concerning the subjective assessment of quality of life in four areas: Sense of Self (14 items), Relationships (14 items), Environment (10 items) and General Quality of Life (3 items). The respondent answers questions on an 11-point scale (from 0—“not at all” to 10—“a great deal or completely”). The items reflect the subject’s perception and evaluation of different aspects of life, thus the raw scores describing subjective quality of life or perceived quality of life in the four areas as well as the total result are obtained (Topolski et al. 2002). Some examples of items are as follows:

I can handle most difficulties that come my way.

I feel I am getting the right amount of attention from my family.

I am happy with the friends I have.

Internal consistency Cronbach’s alpha of the whole scale is α = 0.95, and for particular areas ranges between 0.81 and 0.89. Stability of the scale is *r*_*tt*_ = 0.78 for the total score and for the domains from 0.74 to 0.85.

#### Participants

The research was conducted in Poland on a group of 233 adolescents (148 girls and 85 boys), ranging in age from 16 to 19, who attended high school (mean age *M* = 17.09, *SD* = 0.76). In order to determine identity statuses of participants, median scores for exploration (*Me* = 55) and commitment (*Me* = 62) were used. From 233 participants, 209 adolescents met the criteria which allowed to classify them into one of the four identity statuses (24 persons, whose results in one or another scale—C or P—equaled the median, were excluded). According to the criteria for the four statuses of identity given the level of exploration and commitment processes, diffused (E−C−) met 50 youths (21.5 %), foreclosed (E−C+) *N* = 51 (21.9 %), moratorium (E−C+) 57 (24.4 %) adolescents, and achieved (C+E+) 51 (21.9 %).

In the diffused identity status group, the number of boys and girls was equal, in moratorium identity status group, there were slightly more girls, and in the two remaining groups, there were twice as many girls as boys, which was partly due to the fact that girls formed the majority of all the participants. There was no associations between gender and four statuses of identity, χ^2^ = 4.69, *p* < 0.22, n. s. (see Table 1).

**Table 1:** Gender composition across ego identity statuses

Ego identity status/gender	Diffused *N* (%)	Foreclosed *N* (%)	Moratorium *N* (%)	Achieved *N* (%)	Total *N* (%)
Males	25 (50.0)	17 (33.3)	22 (38.6)	16 (31.4)	80 (38.3)
Females	25 (50.0)	34 (66.7)	35 (61.4)	35 (68.6)	129 (61.7)
Total	50 (100.0)	51 (100.0)	57 (100.0)	51 (100.0)	209 (100.0)

### Statistical Analyses

The relationship between quality of life as perceived by adolescents and identity statuses was investigated by means of the Pearson coefficient test. Five patterns of the connection between identity dimensions and perceived quality of life have been distinguished through cluster analysis. Descriptive analyses included the calculation of means and standard deviations. Differences in the quality of life scores provided by adolescents were compared among groups of adolescents using ANOVA and MANOVA analysis of variance. All statistical analyses were conducted with SPSS software (ver. 17.0; SPSS).

## Results

First, the correlations between the investigated identity dimensions and subjective quality of life were computed. Table 2 presents correlation coefficients.

**Table 2:** Correlations of commitment and explorations scores (EIPQ) with quality of life (YQOL-R)

EIPQ/YQOL dimension	Sense of self		Relationships		Environment		General QOL		Total QOL	
	*r*	*p*<	*r*	*p*<	*r*	*p*<	*r*	*p*<	*r*	*p*<
Commitment	0.11	0.09	0.14	0.05	0.17	0.01	0.14	0.05	0.17	0.02
Exploration	−0.06	n. s.	−0.11	n. s.	0.05	n. s.	−0.06	n. s.	−0.05	n. s.

Low but significant correlations show a weak relationship between commitment and quality of life—in environment, general quality of life, and total score. There are weak positive but statistically significant linkages between commitment identity dimension and total quality of life score, and the three quality of life domains i.e. relationships, environment, and general satisfaction with life. There are low and non significant negative correlations between quality of life and exploration.

**Fig. 1:**
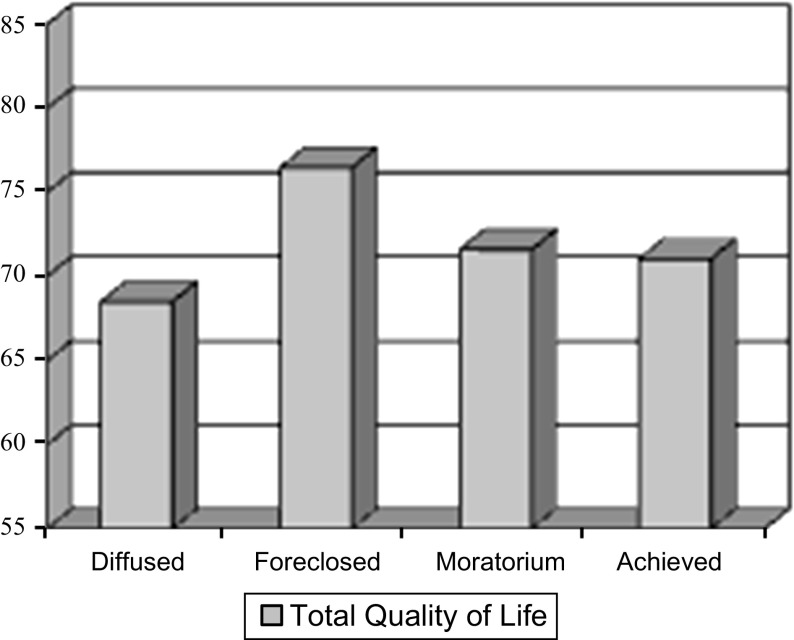
Level of subjective quality of life in four identity status groups

Next, the level of subjective quality of life was analyzed in the identity status groups of adolescents (see Table 3; Fig. 1).

**Table 3:** Comparison of four identity status groups in subjective quality of life: ANOVA and MANOVA

Identity status group/QOL domain	Diffused (*N* = 50)		Foreclosed (*N* = 51)		Moratorium (*N* = 57)		Achieved (*N* = 51)		Differences	
	*M*	*SD*	*M*	*SD*	*M*	*SD*	*M*	*SD*	*F*(3,205)	*p*<
Sense of self	67.27	13.48	71.75	13.99	67.53	16.42	66.11	14.89	1.43	n. s.
Relationships	65.4	14.25	73.30	15.60	68.43	16.79	67.28	16.34	2.57^a^	0.05
Environment	73.50	11.82	78.69	11.63	76.14	14.25	76.96	12.03	1.50	n. s.
General QOL	67.93	26.26	81.76	19.26	75.20	23.64	73.73	22.79	3.05^a^	0.03
Total QOL	68.52	14.21	76.47	12.87	71.60	16.12	71.05	13.73	2.72^a^	0.05
MANOVA: *F*(4,202) = 1.43, *p* < n. s.										

^a^Significant difference between foreclosed and diffused, *p* < 0.05

The four identity status groups differ as far as total quality of life is concerned that is the sum of the results in the four domains [ANOVA: *F*(3,205) = 2.72, *p* < 0.05]. The participants in foreclosed status declare the highest level of total quality of life, while those in the diffused status—the lowest. Nevertheless, the distinguished groups do not differ significantly considering the overall structure of quality of life in the four domains [MANOVA: *F*(4,202) = 1.43, n. s.].

The above results show statistically significant differences concerning only the foreclosed and diffused identity status groups. Those in foreclosed status are characterized by a significantly higher quality of life in the areas of satisfaction with life and social relations than those in the diffused status. There are no significant differences concerning quality of life among groups in achieved, diffused and moratorium identity statuses.

**Fig. 2:**
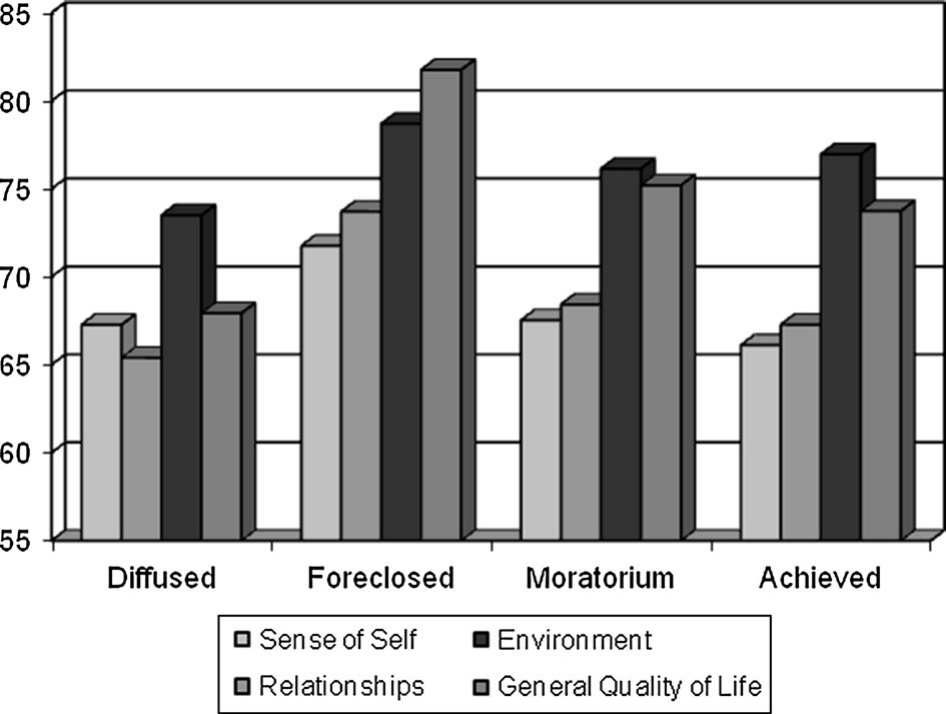
Quality of life in QOL domains in four identity status groups

In addition to the differences between the groups, it is important to emphasize that further disparities appear within the groups themselves: in the areas of quality of life, higher results can be found in the environment domain in all four groups, and lower results in such domains as sense of self and satisfaction with social relations in the groups of diffused, moratorium and achieved status (see Fig. 2). Regardless of exploration and commitment results, the adolescents are characterized by a lower sense of one’s own self, self-confidence, and self-esteem, as well as by a lower level of satisfaction with their physical and mental health, and spiritual dilemmas. Similarly, a lower level of satisfaction can be identified in the field of social relations which, in turn, may indicate not very satisfying relationships with others—especially with family and peers. The participants from the three groups are sufficiently satisfied with the opportunities they have in their communities: education, activity, financial resources and perspectives for the future respectively.

The division based on identity status did not, however, differentiate the participants as regards the feeling of quality of life in a clear-cut way. In order to find more uniform groups for the two identity processes and the global indicator of quality of life, cluster analysis, using k-means clustering algorithm, was performed. The solution with five clusters was selected for the interpretation; for results, see Table 4.

**Table 4:** Comparison between five cluster groups in commitment, exploration and total quality of life: ANOVA

Cluster/dimension	1 (C+E−) (*N* = 64)		2 (C−E−) (*N* = 22)		3 (C+E+) (*N* = 30)		4 (C−E−) (*N* = 44)		5 (C−E+) (*N* = 73)		Differences	
	*M*	*SD*	*M*	*SD*	*M*	*SD*	*M*	*SD*	*M*	*SD*	*F*(4,228)	*p*<
Commitment	69.98	5.73	57.86	6.23	62.80	7.72	60.32	6.08	57.11	5.42	42.92	0.001
Exploration	51.50	7.57	52.73	6.00	64.80	4.82	51.05	5.50	58.97	7.04	31.83	0.001
Total QOL	81.84	6.47	43.57	8.16	57.7	8.77	64.45	4.94	81.37	5.95	225.46	0.001

Five groups of persons display some similarities to the four groups determined on the basis of identity dimensions. Correspondingly, the isolated cluster groups are described in terms of identity dimensions. Cluster 1 represents a higher level of commitment and a lowered level of exploration, which indicates foreclosed identity (C+, E−) as conceptualized by Marcia (1966). Cluster 2, the least numerous group, and cluster 4, with twice as many participants, represent a lowered level of both commitment and exploration, which suggests diffused identity (C−, E–)—a similar identity pattern in both groups, in contrast to a completely different level of quality of life. A higher level of commitment and exploration among participants in cluster 3 indicates achieved identity (C+, E+), while moratorium identity (C−, E+) emerges in cluster 5 with a lower commitment and higher exploration. These groups differ very significantly (*p* < 0.001) in both identity dimensions and in the global indicator of perceived quality of life [ANOVA: *F*(4,228) = 225.46, *p* < 0.001].

The highest indicator of subjective quality of life appears in cluster 1 (C+E, foreclosed identity), and cluster 5 (moratorium). The lowest indicator appears in cluster 2 (diffused), while cluster 3 (achieved) is characterized by a significantly lower quality of life than in cluster 5 (moratorium) and cluster 4 (diffused) but by a higher quality of life than cluster 2 (also diffused).

Groups 1 and 5 are marked by a similar, relatively high level of subjective quality of life. However, persons from group 1 (foreclosed) demonstrate commitment without asking themselves identity questions. They adopt the rules from important people without questioning them. These persons do not take up identity issues but they are satisfied and have a relatively high feeling of quality of life. Adolescents from group 5, on the other hand, still have unsolved identity issues and they are at the stage of testing different possibilities. Moreover, in spite of their lack of commitment to realize goals and values, they have a relatively high feeling of quality of life. They prefer changeability and treat life like an experiment, without making final decisions, which coexists with a relatively high feeling of quality of life. A surprisingly low feeling of quality of life is characteristic of adolescents with achieved identity—cluster 3. Searching for answers to identity questions and commitment to pursue the chosen values does not ensure a proper feeling of quality of life. Once formed identity may lead to the necessity of renouncing many attractive forms of activity, excluding or limiting new challenges, or, on the other hand, it may be connected with a fear of change.

In group 1, the level of commitment is higher than in all other groups; in comparison to group 3 and 5, the level of exploration is lower, but quality of life is higher than in all groups except group 5, where it is comparatively high. In the second group, the feeling of quality of life is definitely the lowest, commitment is also lower in comparison to groups 1 and 3, and exploration is significantly less intensive in comparison to groups 3 and 5. The third group is characterized by the highest level of exploration, lower commitment than group 1, but significantly higher than groups 2 and 5, and relatively low quality of life, lower than in groups 1, 4 and 5, and higher only than in group 2. Group 4 (similarly to group 1) is characterized by a low level of exploration, significantly lower than in groups 3 and 5, a higher quality of life in comparison to groups 2 and 3 but lower than groups 1 and 5, with commitment higher than group 5 but lower than group 1. Group 5 (similarly to group 1) has high results in quality of life, significantly higher in comparison to groups 2, 3 and 4, and low (similarly to group 2) commitment, significantly lower in comparison to groups 1, 3 and 4. In the dimension of exploration, the result of group 5 is significantly higher than the results from groups 1, 2 and 4, and lower than group 3. Table 5 presents the distribution of significant differences in identity dimensions and total quality of life between the cluster groups.

**Table 5:** Significant differences in identity dimensions and total quality of life between the cluster groups

	1 (C+, E−)	2 (C−, E−)	3 (C+, E+)	4 (C−, E−)	5 (C−, E+)
2	C** QOL**		C* E** QOL*	QOL**	E** QOL
3	C** E** QOL**	C* E** QOL*		E** QOL**	C** E** QOL**
4	C** QOL**	QOL**	E** QOL**		C* E** QOL**
5	C** E**	E** QOL**	C** E** QOL**	C* E** QOL**	

*C* commitment, *E* exploration, *QOL* quality of life

* Differences significant on *p* < 0.05

** Differences significant on *p* < 0.001

There are significant differences between the distinguished groups regarding both the domains of quality of life (ANOVA) and the four domains altogether [MANOVA: *F*(4,16) = 57.16, *p* < 0.001]. See Table 6.

**Table 6:** Comparison between five cluster groups in quality of life domains: ANOVA and MANOVA

Cluster/quality of life domains	1 (*N* = 64)		2 *N* = 22)		3 (*N* = 30)		4 (*N* = 44)		5 (*N* = 73)		Differences	
	*M*	*SD*	*M*	*SD*	*M*	*SD*	*M*	*SD*	*M*	*SD*	*F*(4,228)	*p*<
Sense of self	76.71	8.77	46.30	10.62	54.24	11.05	60.70	8.63	76.87	9.86	78.86	0.001
Relationships	78.08	9.81	40.74	11.60	54.45	14.14	61.91	9.33	77.33	8.63	87.06	0.001
Environment	82.70	7.81	56.50	12.48	68.43	10.99	69.75	9.91	81.99	8.90	47.97	0.001
General QOL	81.22	10.37	30.76	14.98	57.78	20.80	66.59	14.93	89.27	8.89	126.84	0.001
MANOVA: *F*(4,225) = 31.14, *p* < 0.0001												

Cluster 1 (foreclosed identity status) and cluster 5 (moratorium) display a similar level of subjective quality of life in all four areas. Hence, it can be argued that persons with opposite identity processes of exploration and commitment—i.e. those not undergoing the period of exploration but committed to realizing a priori adopted standards, and persons intensely seeking—are characterized by general satisfaction with life, with themselves, with their social relations and with the possibilities offered by the environment. A lower satisfaction with life, with themselves, and with their interpersonal relations is characteristic of adolescents with achieved identity (cluster 3). Clusters 2 and 4 (diffused identity status) reveal a different intensity of subjective quality of life in the four domains: cluster 2—a very low level of perceived quality of life, that is frustration regarding a general assessment of life, sense of one’s own self, relations with others and satisfaction with the environment; cluster 4—a significantly higher feeling of quality of life in all these domains, although lower in comparison to persons from cluster 1 (foreclosed identity) and 5 (moratorium). This is interesting that these persons do not take up challenges connected with their own identity formation either. What is more, cluster 3 (achieved) reveals a significantly lower feeling of quality of life than persons from cluster 4 (diffused) in three out of four areas of quality of life—in the area of sense of one’s own self, social relations and general satisfaction with life.

Group 1 represents a considerably higher feeling of quality of life in each of the spheres, in comparison to the other groups, except for group 5 where the level of quality of life feeling is similar. Definitely, the lowest feeling of quality of life in all the areas in comparison to all the other groups is characteristic of persons from group 2. Group 2 has the lowest level of general life satisfaction in particular spheres. Group 3 differs from groups 1 and 5 in a lower quality of life feeling in all the areas; it differs from group 4 in a lower general satisfaction with life, with oneself and with social relations, and it is different from group 2 in displaying a higher satisfaction with social relations and environmental opportunities as well as higher satisfaction with oneself. Group 4 is characterized by a lower level of quality of life feeling in all the domains in comparison with groups 1 and 5 but higher satisfaction with oneself, with social relations and general life satisfaction than groups 2 and 3. Quality of life in environment is as low in this group as in group 3 but it is higher than in group 2. The achieved results demonstrate the complexity of identity phenomena in adolescents as well as an ambiguous connection between identity processes and subjective quality of life. The general comparison of significant differences between the clusters in quality of life domains is presented in Table 7.

**Table 7:** Significant differences between the clusters in quality of life domains

Cluster	1	2	3	4
2	SelfDom**			
	RelDom**			
	EnvDom**			
	GenDom**			
3	SelfDom**	SelfDom*		
	RelDom**	RelDom**		
	EnvDom**	RelDom**		
	GenDom**	GenDom**		
4	SelfDom**	SelfDom**	SelfDom*	
	RelDom**	RelDom**	RelDom*	
	EnvDom**	**EnvDom** **	GenDom**	
	GenDom**	GenDom**		
5		SelfDom**	SelfDom**	SelfDom**
		RelDom**	RelDom**	RelDom**
		EnvDom**	EnvDom**	EnvDom**
		GenDom**	GenDom**	GenDom**

*SelfDom* sense of self, *RelDom* relationships, *EnvDom* environment, *GenDom* general quality of life

* Differences significant on *p* < 0.05

** Differences significant on *p* < 0.001

## Discussion

The intention of this study is to verify the hypotheses concerning identity processes and subjective quality of life in adolescents. In view of the research results presented above, the first of the hypotheses postulating a negative correspondence between exploration and subjective quality of life and a positive correspondence between commitment and quality of life has been partly confirmed. Commitment positively corresponds—however weakly—with subjective quality of life. In his study of the connection between commitment and well-being, Berzonsky (2003), underlines the significance of different types of commitment for the improvement of well-being and everyday activity. Commitment is connected with the choice of profession, religion, opinions, way of behaving in interpersonal relations, etc., which may correspond to the global feeling of quality of life. Marcia (1989) also highlights the connection between identity and well-being. According to him, an achieved identity pattern is the healthiest as it indicates the individual’s ability to adapt to their surroundings.

The first hypothesis has not been confirmed as far as exploration is concerned. Testing new tasks, roles, goals and values is not connected to subjective quality of life even though the ability to explore the environment in the fast-changing and quite unpredictable social environment is a basic ability and an important condition of health (Archer 1989).

The second hypothesis, postulating that adolescents with achieved identity are characterized by the highest level of subjective quality of life, while persons with diffused identity are characterized by the lowest feeling of quality of life, has also been partly confirmed. The analysis of the results from the four groups with different identity statuses proves that adolescents with diffused identity pattern represent the lowest level of quality of life, while the highest quality of life level is characteristic of persons with foreclosed identity, and not with achieved identity status as was previously assumed. It seems that in the case of persons with diffused identity there are no definite preferences or stable self-determination, whereas immediate pleasure drawn from different types of activities does not bring a long-term satisfaction, nor does it provide the ground for a high evaluation of quality of life. Yet, a relatively high level of quality of life in persons with foreclosed identity may mean that for many people in adolescence foreclosed identity is highly adaptive.

According to Meeus et al. (1999) persons at foreclosed identity stage adopt standards from the surrounding environment without making the effort to solve problems, choose goals, values, roles or beliefs concerning the surrounding world. As they are not affected by identity dilemmas, they maintain well-being. The lowest indicator of quality of life is characteristic of persons at the stage of identity diffusion who have not yet made choices concerning important values in their lives and so have not been involved in realizing them (Meeus et al. 1999).

The lowest level of well-being is identified in persons in moratorium, owing to the “costs” of identity crisis—related to intensive exploration and low commitment. In the case of diffused identity pattern, low commitment is not so destructive due to the fact that the level of exploration is also low. Nevertheless, in the stages of strong commitment—foreclosed and achieved—the level of exploration is of no significance for well-being.

The interpretation of the results may be conducted in reference to the three identity styles: informative, normative and diffuse/avoidant orientation. The least mature style, which correlates negatively with well-being, is used by persons with diffused identity. Those at the stage of identity foreclosure, however, because of commitment, use normative style, yet its connection to well-being has not been conclusively demonstrated so far (Vleioras and Bosma 2005). Moreover, as the research conducted by Berzonsky (1992) shows, persons with diffuse and normative styles use non-adaptive coping strategies: wishful thinking, distancing or reducing tension. It is probable that by staying on the “safe side” and avoiding risky decisions or actions, persons with foreclosed identity gain a sense of confidence and safety which results in well-being. Risky behaviour (drugs, crime) does not coexist with foreclosed identity connected to conservative attitudes (King, in: Dunkel and Papini 2005, p. 490).

The hypothesis postulating a higher quality of life in persons with achieved identity has not been confirmed. However, if we assume that the lowest level of development is associated with the diffused identity stage, persons, in whom the processes of exploration and commitment are not present, also reveal the lowest level of subjective quality of life (with both criteria of group division). This is consistent with the results of the research conducted so far (Meeus et al. 1999; Vleioras and Bosma 2005). At the same time, there are persons with diffused identity who are characterized by a higher feeling of quality of life than those with achieved identity—an unexpected result revealed in cluster analysis. The highest indicator of subjective quality of life, on the other hand, is characteristic of participants with exploration without commitment or commitment without exploration (moratorium and foreclosed identity), not of those with achieved identity. A possible explanation of this intriguing phenomenon is that there are two patterns coexisting in culture. One, favouring multiplicity and variety of experiences—“a postmodernist style of functioning”—moratorium, therefore, offers an opportunity for a relatively high quality of life. The other—“traditional style”—where one is deeply grounded in specific beliefs, values or behaviour patterns that correspond to foreclosed identity and are connected with a relatively high quality of life. The former can be explained in the following way: the person with unstable evaluating criteria has a chance for a more exciting life (Arnett 2002), while the latter suggests that people, who do not make the cognitive effort of self-determination and of choosing their values, do not experience doubts, dilemmas, uncertainty or anxiety. A high level of well-being in the foreclosed identity group is confirmed by the research conducted by Meeus et al. (1999). It is possible that the lack of adequate abilities—of critical thinking, problem solving, changing of point of view and value orientation—causes one to strictly follow accepted values (Grotevant 1987). It minimizes the risk of bearing the costs of exploration.

Among the participants, there are persons who do not take up identity challenges (one of the groups with diffused identity) and are characterized by a higher level of quality of life than the participants who practice exploration, look for a place for themselves, test themselves in various roles, make important decisions and realize chosen values (achieved identity). Could this mean that achieved identity is not the highest and most desired stage of development? Or that this stage of development is not connected with high well-being and subjective quality of life nowadays? It might well be that, at present, new identity formation rules are developing, going beyond classifications proposed in the middle of the twentieth century. Cultural transformations create a pressure more in the direction of forming a fluid identity rather than the final solution of identity dilemmas i.e. stable, achieved identity (Amiot et al. 2007). On the one hand, there is a striving for self-definition, on the other, questioning identity. Achieved identity is connected with a choice of particular lifestyle and preferences, a choice which—in view of such a broad cultural offer—requires selective commitment but, above all, requires giving up a number of other attractive possibilities.

Higher quality of life in the group with diffused identity, in comparison to the group with achieved identity, may indicate that some young people function in an adaptive manner, not making permanent choices and decisions, and adopt identity only on a temporary, trial basis, without exploration. They make no effort to search for identity, but instead they pick adaptive behaviours for different occasions.

The third hypothesis, postulating differences of perceived quality of life among persons characterized by a different pattern of identity processes and quality of life together, has been confirmed to a large extent. Different patterns of identity processes are related to diverse levels of subjective quality of life. The differences are compatible with the results obtained previously as far as diffused identity is concerned; however, some unexpected results have been achieved as regards achieved identity and moratorium. A higher quality of life in achieved identity by comparison to diffused identity conforms to the hypothesis, yet a higher of quality of life in moratorium than in achieved identity does not.

This may indicate that identity processes are dynamic and develop in a multi-faceted way, while identity patterns seldom remain the same forever. The research results lead us to the conclusion that identity formation should be understood in a much broader perspective than it has been so far. Globalization makes identity phenomena complex not only in a cultural aspect. The area of identity issues has broadened significantly, from more traditional like occupation, politics, and religion, to more progressive including friendship, dating, or gender roles (Arnett 2002). Another question that concerns the very process of identity formation, which nowadays takes so much longer, is the context in which different solutions of identity issues appear (Kroger 2000). Cultural transformations and new challenges, which the adolescents struggle with, create a wider space for testing different roles and value systems without the necessity of making binding decisions (see e.g. Lichtwarck-Aschoff et al. 2008). Hence, further research should focus more on factors connected with the development, stability and regression of identity. There is also a need for a more insightful study of the phenomena defined as moratorium, especially from the perspective of social context, that exerts such an influence on young people’s identity formation.

The dynamic approach to identity, suggested now, is based on the assumption that adolescents constantly reflect upon their commitment (Meeus et al. 1999). It is not the case, then, that exploration is important mainly in the choice of commitment after which it may no longer be taken into account (Marcia 1966). Identity formation is a long-running process which does not lead to a final solution in the form of an achieved, stable identity. For this reason, a processual approach to identity is proposed in order to supersede a restricted, rigid and dated approach drawing on stable statuses (Côté and Levine 1988). It should be emphasised that even achieved identity may change later in life (Sneed et al. 2006; Stephen et al. 1992; Kroger et al. 2010). Identity formation is dynamic—a constant identity struggle requiring a continuous effort. On the one hand, there is a tendency for self-determination, on the other, for changing and reforming identity (Amiot et al. 2007; Arnett 2002; Kunnen et al. 2001a). Identity should be seen as “…rooted in emotion, emerging in relationships, and developing as a dynamic, self-organizing system…” (Kunnen et al. 2001b, p. 5). Thus, the subjective quality of life in adolescents could also be subject to high alteration and changeability.

## Footnotes

1 In this study, the terms ‘quality of life’, ‘subjective quality of life’ and ‘perceived quality of life’ are used as interchangeable concepts to convey the subjective character of these variables. A person’s quality of life refers to their perceived physical, psychological, social and spiritual well-being over time.
